# Acute Appendicitis Presenting As Left Flank Pain: A Case Report

**DOI:** 10.7759/cureus.62181

**Published:** 2024-06-11

**Authors:** Ibrahim Badawy, Khaled Mbaya, Hesham Metwally

**Affiliations:** 1 Urgent Care Department, Mediclinic Hospital, Al Ain, ARE; 2 Radiology Department, Mediclinic Hospital, Al Ain, ARE

**Keywords:** case report, computed tomography, atypical, left flank pain, appendicitis

## Abstract

Acute appendicitis (AA) is a common surgical emergency. The diagnosis is mainly clinical and is largely based on the typical presentation of periumbilical pain radiating to the right iliac fossa. However, atypical presentations have been reported in the literature. Left-sided appendicitis is a rare presentation. Imaging with ultrasonography (US) and computed tomography (CT) is of paramount importance in such cases to aid diagnosis and exclude other causes. We report a case of a 21-year-old man who presented with acute left flank pain. He was diagnosed with AA by CT, which showed an appendix diameter of 13 mm.

## Introduction

Acute appendicitis (AA) is a common surgical emergency. Its main pathology is obstruction of the appendiceal orifice causing progressive distension and inflammation. This distension allows the growth of pathogenic microorganisms, in addition to eventual vascular compromise [1,2].

Typically, appendicitis pain starts in the periumbilical region. Then, it migrates to the right iliac fossa, and it is usually accompanied by anorexia, nausea, and vomiting. The presence of Blumberg's sign, Rovsing's sign, and the Psoas sign can aid the diagnosis of AA. Scoring systems combining clinical signs with inflammatory markers were created to further facilitate the diagnosis and help make treatment plans, among which the Alvarado score system is widely used [3]. Although the diagnosis of AA used to be largely clinical, the role of imaging is becoming more common in attempts to increase the accuracy of diagnosis whenever doubt exists [1].

The vermiform appendix usually lies in the right lower quadrant of the abdomen. However, its tip can be found in various sites including retrocaecal, paracaecal, subcaecal, pelvic, pre-ileal, and post-ileal regions [4]. These variations can cause atypical pain presentations that may confuse the diagnosis. Left-sided appendicitis is a very rare presentation and is usually associated with a left-sided appendix as a part of a congenital anomaly as situs inversus or midgut malrotation, which was estimated to occur in one of 6000 live births [5,6]. The problem that arises is the differential diagnosis of left-sided abdominal pain. Several conditions can cause this pain including diverticulitis, kidney stones, inflammatory bowel disease, irritable bowel syndrome, and constipation. This compromises the role of clinical examination in diagnosing AA. Even scoring systems in this setting might not be able to identify appendicitis. For example, Alvarado assigned the highest score (of two) to right iliac fossa tenderness and leukocytosis [3]. Incorrect diagnosis of appendicitis may lead to unnecessary surgery, and delay in diagnosis predisposes the patient to complications including abscess and perforation [7]. That is why imaging such as ultrasonography (US) and computed tomography (CT) can be of great importance in this setting. Here, we report a case of a male patient with acute left-sided flank pain that was eventually diagnosed as AA.

## Case presentation

A 21-year-old male presented to our emergency room complaining of acute onset left flank pain for two hours that was accompanied by nausea. He reported no other symptoms. The patient was vitally stable. Abdominal examination revealed tenderness in the left flank and left lumbar area. The patient had no history of previous similar conditions. He had no current medical or surgical problems. Laboratory investigations showed leucocytosis, whereas renal and liver function tests and electrolyte serum levels were all normal (Table 1).

**Table 1:** Laboratory Investigations of the Case. ALT: alanine aminotransferase; AST: aspartate aminotransferase

Test name	Result	Unit	Reference range
Total leucocytic count	15	Thousands/cmm	5-10
Serum creatinine	0.97	mg/dL	0.5-1.1
Serum urea	41	mg/dL	17-49
ALT	17	U/L	0-33
AST	20	U/L	0-32
Serum sodium	138	mEq/L	135-145
Serum potassium	3.9	mEq/L	3.5-5.5

**Figure 1:**
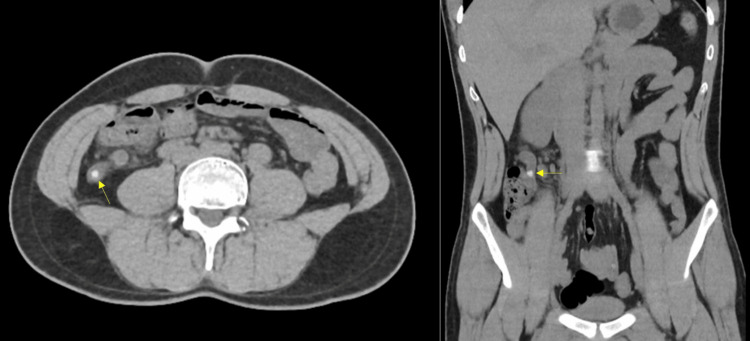
Abdominal CT Showing Left-Sided Retrocecal Appendix With Fecolith (Arrow) and Significantly Increased Diameter. CT, computed tomography

Abdominal US and kidneys, ureters, and bladder radiography did not reveal any stones or back pressure on the kidneys. The report recommended performing pelviabdominal CT because acute diverticulitis was suspected. The patient was given analgesics including paracetamol one gm infusion over 20 minutes and dexketoprofen 50 mg infusion over 20 minutes. Eventually, CT showed signs of AA. The appendix was retrocecal in position with a significantly increased diameter of 13 mm. There was surrounding fat stranding with multiple enlarged mesenteric lymph nodes (Figure 1).

The patient was admitted to the Surgery Department for AA without peritonitis, exhibiting normal peristalsis and no abdominal distension. The surgeon devised a plan of nothing by mouth, intravenous antibiotics, and fluids until the operation. Laparoscopic appendicectomy was performed under general anesthesia. Postoperative follow-up revealed that the patient was experiencing general well-being, with no nausea, vomiting, rebound tenderness, abdominal distension, or guarding. He was able to ambulate, pass gas, and eat solid food one day after surgery. The patient's recovery progressed well, and he was discharged from the hospital two days after surgery. The resected appendix was sent for histopathologic examination. The report showed a vermiform appendix with grey serosa. The lumen was dilated and filled with fecal material, the wall had an average thickness of 0.3 cm, and no discrete mass was identified.

## Discussion

We report a unique case of AA with the rare presentation of acute left flank pain. Left-sided pain as the presenting symptom of AA is rare. It is usually encountered when the appendix is truly on the left side of the abdomen as in situs inversus or midgut malrotation [8]. Yang et al. reported a case of left-sided appendicitis that was explained by the hypermobility of the ascending colon that led the cecum to move to the left side of the abdomen [9]. Appendicitis presenting with left flank pain has only been reported once in the literature [10].

The atypical pain site represents a diagnostic challenge in AA. Left flank pain in a male patient has a wide range of differential diagnoses including diverticulitis, kidney stones, inflammatory bowel disease, irritable bowel syndrome, and constipation. All these conditions were considered when approaching our patient. Since clinical examination alone was not conclusive, laboratory investigations and imaging were necessary. In spite of the controversy regarding the role of the US in AA diagnosis, in cases of doubt, it may aid the diagnosis, detect other underlying pathologies, and help narrow down the differential diagnosis [3]. Unfortunately, in our case, the US was normal prompting further imaging to reveal the real etiology. Usually, physicians only resort to CT when the US is inconclusive to avoid unnecessary radiation. Our patient represents one of those cases where CT was of great importance in early diagnosis. CT in our patient was diagnostic of AA showing an appendiceal diameter of 13 mm (AA is diagnosed when the diameter exceeds 6.5 mm), in addition to inflammation of the peri-appendiceal fat. A dilated, fluid-filled appendix is the most specific CT finding [11]. Other reported signs are arrowhead sign, cecal bar sign, and the presence of appendicolith [2,12]. Talanow reported a case with a similar presentation to ours [10]. Non-contrast CT revealed only an appendicolith, yet the appendix was not dilated. A week later, the patient presented with a perforated appendix. That is why Talanow concluded that an appendicolith in a patient with left flank pain with no evidence of urolithiasis may be a sign of early appendicitis.

## Conclusions

We report a case of a 21-year-old male with AA presenting as acute left flank pain. Our case highlights the importance of including AA in the differential diagnosis of acute abdomen even if the pain is not in the typical site. It also shows the importance of CT imaging in doubtful diagnosis, especially with inconclusive US. Early diagnosis of AA is important to avoid unnecessary complications including perforation.
